# Fine-grain population structure and transmission patterns of *Mycobacterium tuberculosis* in southern Mozambique, a high TB/HIV burden area

**DOI:** 10.1099/mgen.0.000844

**Published:** 2022-07-05

**Authors:** Belén Saavedra Cervera, Mariana G. López, Álvaro Chiner-Oms, Ana María García, Irving Cancino-Muñoz, Manuela Torres-Puente, Luis Villamayor, Carlos Madrazo-Moya, Edson Mambuque, Guillermo Victor Sequera, Durval Respeito, Silvia Blanco, Orvalho Augusto, Elisa López-Varela, Alberto L. García-Basteiro, Iñaki Comas

**Affiliations:** ^1^​ PhD Programin Medicine and Translational Research, Universitat de Barcelona, Barcelona, Spain; ^2^​ Centro de Investigação em Saúde de Manhiça (CISM), Maputo, Mozambique; ^3^​ ISGlobal, Hospital Clínic – Universitat de Barcelona, Barcelona, Spain; ^4^​ Instituto de Biomedicina de Valencia (IBV), CSIC, Valencia, Spain; ^5^​ Universidad de Valencia, Valencia, Spain; ^6^​ FISABIO Public Health, Valencia, Spain; ^7^​ Centro de Investigación Biomédica en Red de Enfermedades Infecciosas (CIBERINFEC), Barcelona, Spain; ^8^​ CIBER in Epidemiology and Public Health, Madrid, Spain

**Keywords:** genomics, transmission, molecular epidemiology, *Mycobacterium tuberculosis*, tuberculosis

## Abstract

Genomic studies of the *Mycobacterium tuberculosis complex* (MTBC) might shed light on the dynamics of its transmission, especially in high-burden settings, where recent outbreaks are embedded in the complex natural history of the disease. To this end, we conducted a 1 year prospective surveillance-based study in Mozambique. We applied whole-genome sequencing (WGS) to 295 positive cultures. We fully characterized MTBC isolates by phylogenetics and dating evaluation, and carried out a molecular epidemiology analysis to investigate further associations with pre-defined transmission risk factors. The majority of strains (49.5%, 136/275) belonged to lineage (L) 4; 57.8 % of them (159/275) were in genomic transmission clusters (cut-off 5 SNPs), and a strikingly high proportion (45.5%) shared an identical genotype (0 SNP pairwise distance). We found two ‘likely endemic’ clades, comprising 67 strains, belonging to L1.2, which dated back to the late 19th century and were associated with recent spread among people living with human immunodeficiency virus (PLHIV). We describe for the first time the population structure of MTBC in our region, a high tuberculosis (TB)/HIV burden area. Clustering analysis revealed an unforeseen pattern of spread and high rates of progression to active TB, suggesting weaknesses in TB control activities. The long-term presence of local strains in Mozambique, which were responsible for large transmission among HIV/TB-coinfected patients, calls into question the role of HIV in TB transmission.

## Data Summary

The authors confirm all supporting data, code and protocols have been provided within the article or through supplementary data files.

**Impact Statement**

This is the first report presenting a comprehensive molecular epidemiology analysis, by combining results on fine-scale population structure, transmission profiles and sociodemographic variables in southern Mozambique, a high TB/HIV burden setting. Information from a 1 year population-based TB surveillance study was combined with whole-genome sequencing data. We fully characterized *Mycobacterium tuberculosis complex* (MTBC) strains using different phylogenetic approaches and dating evaluation. We also described the transmission profile, and explored possible associations with host and pathogen factors. We described ‘likely endemic’ clades that were estimated to have been circulating in the country for more than a century, and which were responsible for recent transmission among people living with HIV (PLHIV) (despite early notions that PLHIV are low transmitters). This has a straightforward impact on local TB control but also on our understanding of HIV/TB and host interplay. We additionally reported an unexpected proportion of strains involved in recent transmission, which sheds light on the extent of the epidemic and suggests that ongoing public health interventions are not sufficient to control the spread of MTBC.

## Introduction

Tuberculosis (TB) remains one of the deadliest and most prevalent infectious diseases worldwide [1]. More than 20 years after the introduction of molecular tools in TB, it is now undoubted that unravelling the transmission dynamics of local epidemics is essential to tackle ongoing spread [2]. A complete understanding of who transmits, where, how and why, is essential for designing effective control interventions [3].

The development of whole-genome sequencing (WGS) techniques is transforming the classical vision of the infection and decoding links unreachable by basic epidemiology or traditional genotyping [4]. However, WGS is still rarely applied in high-burden settings, where recent outbreaks are embedded in the complex natural evolution of the disease [5].

High-resolution genomic data allow for accurate cluster analysis to investigate outbreaks and decrypt transmission profiles. By using the single nucleotide polymorphism (SNP) pairwise distance among strains, we can delineate transmission clusters with a resolution not seen before [4]. Additionally, the study of the origin and genetic diversity of local strains also helps to survey the evolutionary history of the *Mycobacterium tuberculosis complex* (MTBC) population and the potential variations between lineages [5, 6].

Phylogeographical methods have identified different lineages and sub-lineages of MTBC with differential distributions worldwide [7]. This striking difference in geographical location has led to hypotheses as to why some are more widespread than others. Whereas Lineage (L) 4 is the most ubiquitous globally, L1 and L3 have been described as endemic to several high-burden regions in Asia and Eastern Africa [8]. Although part of those differences can be explained by historical contingency [9], host–pathogen coevolution is assumed to play a decisive role [10]. In San Francisco, there seemed to be a preference for transmission between lineages and hosts with the same geographical origin [11]. Furthermore, some studies suggest that this link between human populations and lineages from the same geographical origin breaks down with human immunodeficiency virus (HIV) status, suggesting a role for coevolution at the immune system level [11]. However, identifying why a genotype succeeds locally remains a challenge and there is limited information on the interaction with other factors, such as HIV infection.

The insights we are gaining from WGS data should be transferred to high-burden settings and used to define effective public health interventions. Mozambique is a country with one of the highest rates of TB and HIV/TB coinfection [1], but information on MTBC population structure is scarce [12, 13]. To our knowledge, this is the first comprehensive population-based study in Mozambique on the application of WGS to characterize local strains, unravel likely transmission links, and evaluate possible associations with host and pathogen factors.

## Methods

### Study design and study population

This was a 1 year prospective surveillance-based study (TOSSE study: Tuberculosis observational study: surveillance and epidemiology) implemented from August 2013 to 2014, embedded in the routine TB patient management procedures of the National TB Control Programme. It was conducted in the District of Manhiça, Maputo province, a semi-rural area in southern Mozambique, 80 km north of the capital, with an estimated population of 186,241 inhabitants at the time of the study (2013–2014) [14], living in an area of 2373 km² [15]. This is high TB/HIV burden area with a long history of high TB transmission [16, 17].

The identification of patients was based on the National TB programme (NTP) activities. Therefore, adults with presumptive TB, without a history of previous TB treatment, presenting with TB-compatible symptoms [18] (no time criteria if HIV-positive), and who attended any of the health units belonging to Manhiça District Hospital’s catchment area, were consecutively enrolled [19]. Only participants who started treatment with confirmed MTBC in Xpert MTB/RIF were included in the analysis.

### Diagnostic procedures

Participants provided two sputum samples at the time of diagnosis. Extra-pulmonary specimens were collected at the hospital level. Diagnostic tests were performed at the Centro de Investigação em Saúde de Manhiça (CISM) – Biosafety level 3 (BSL3) laboratory, which is subject to external quality control and is ISO certified.

#### Ziehl-Neelsen (ZN) staining

Smear microscopy was done by ZN staining. Results were reported as negative or on a scale of positive grades according to international standards [20].

#### Xpert MTB/RIF (Xpert)

Raw samples were tested according to the manufacturer's instructions [21]. Invalid results were excluded. Semi-quantitative results for Xpert fell under the following categories: very low, low, medium or high.

#### Solid and liquid culture

Samples with positive Xpert results were cultured. Remaining raw samples were decontaminated by the Kubica method [22] and resuspended. Afterwards, 500 µl was inoculated into Mycobacteria Growth Indicator Tubes (MGIT) liquid medium and incubated in the Bactec MGIT 960 mycobacterial detection instrument (Becton Dickinson Microbiology System). Additionally, 200 µl was cultured in BD Lowenstein Jensen solid medium. After 42 days (for liquid culture) or 8 weeks (for solid culture) without growth, samples were classified as negative. In the case of positive results, MTBC was confirmed using ZN staining and BD TB Identification test (Becton Dickinson Microbiology System). Isolates were stored at −80 °C.

### Sequencing library construction and bioinformatics pipeline

Culture isolates were shipped to the BSL3 laboratory of the Instituto de Biomedicina de Valencia (Spain). After inactivation, samples were used to prepare WGS libraries. Genomic libraries were constructed with the Nextera XT Sample Preparation kit (Illumina) according to the manufacturer’s protocol [23], with 12 cycles for indexing PCR. WGS was carried out in the MiSeq platform (2×300 cycles paired-end run; Illumina).

Sequence analysis was performed following a validated, previously described bioinformatics pipeline [24]. Briefly, FASTQ files were pre-processed with *fastp* [25] to trim poor quality bases and potential sequencing errors. Later, to reduce probable contaminant reads, we classified and filtered those that did not belong to the MTBC using Kraken [26]. Samples with less than 90 % of MTBC reads were discarded for posterior analyses. After this filtering, reads were mapped against the MTBC most probable ancestral genome [27] using the BWA-mem algorithm [28]. Later, we discarded reads with ambiguous mapping based on the BWA MAPQ score (keeping those with MAPQ=60) as well as potential duplicate reads by using *picard* tools. Samples with genomic coverage <90 % were discarded. Variant calling was performed by a combination of SAMtools and VarScan [29].

To avoid mapping errors and false SNPs, we kept variants that (i) were supported by at least 20 reads, (ii) were found at a frequency of at least 0.9, (iii) were not found inside detected indel areas or (iv) were not found in areas of high accumulation of variants (more than three variants in a 10 bp defined window). Variants were annotated using SnpEff [30]. Variants present in PE/PPE genes, phages or repeated sequences were not considered. With these high-quality variants detected, we generated the alignment. Samples with at least two phylogenetic variants at a frequency >10 % were classified as mixed infection cases.

### Phylogenetics and geographical analysis

A maximum-likelihood phylogeny was constructed with IQ-TREE [31] under the General Time Reversible (GTR) model of evolution, with a bootstrap of 1000 replicates and using the *-fconst* option to account for invariant sites. Known drug-resistant positions were not considered for generating the phylogeny as they are highly homoplastic. Later, we aimed to define the population structure of closely related strains by using the fast-hierarchical Bayesian Analysis of Population Structure (BAPS) algorithm implemented in the R library *fastbaps*. BAPS groups were defined under the second level of clustering hierarchy [32].

### Geographical origin of the clades from Mozambique

To unravel the potential geographical origin of the BAPS groups, we reconstructed a phylogeny combining isolates from Mozambique and 8,263 genomes representative of the MTBC global genetic diversity. We marked Mozambique strains as having their geographical origin in Mozambique, and strains from other datasets as having a non-Mozambique origin. After plotting the BAPS groups into the global phylogeny, we used the RASP program [33]. This is based on both, Bayesian and parsimony approaches, and aims to estimate ancestral geographical origins. It requires a phylogeny and the geographical origin of all the tips as input. We coupled the Statistical-Dispersal Vicariance Analysis (S-DIVA) with a Bayesian Binary Markov chain Monte Carlo (MCMC) approach. For each analysis, we ran five MCMC chains with 500,000 cycles. RASP output estimated the likely origin of ancestral nodes.

Lastly, we defined ‘likely endemic’ clades. The characteristics we established to define a BAPS group as 'likely endemic' were as follows: (i) the BAPS group had to be prevalent at the place of interest (this meant that the contribution of Mozambican samples had to be >60 % of the group when pooled with a global collection of samples); and (ii) the likely origin of the most recent common ancestor (MRCA) of the BAPS group obtained with RASP had to be the place of interest (Mozambique >80 % of probability). Once defined, if two or more endemic BAPS grouped together in the phylogeny, then they were merged as a single endemic clade. Non-endemic clades were defined as those in which (i) the strains constituted <60 % of the total group, and (ii) the MRCA was not Mozambican. Unknown clades were those which did not meet any of the previous criteria.

### Dating analysis

Dating analysis was performed by applying the Bayesian inference implemented in BEAST2 v2.6.5 [33]. A multifasta file was generated for the three major lineages (L1, L2 and L4). We used them as partitions in *beauti*. A GTR substitution model was defined with gamma count=4 and empirical nucleotide frequencies. A strict clock model was selected; since we do not have tip or node calibration, we fixed different clock rates for each lineage (L1 : 1.57×10^−7^; L2: 4.1×10^−8^; L4: 3.79×10^−8^), according to Menardo *et al*. [34]. We evaluated exponential and constant coalescent population models. The constant population model is a specific case of the exponential growth model, when the growth rate is equal to zero. Since the 95 % highest posterior density interval (HPD) of the growth rate does not include zero, we can conclude that the data reject a demographic model with constant population size [34]. Thus, we selected the exponential model.

We use exponential priors for the population size (mean=1, offset=0), and for the exponential growth rate prior, we used the standard Laplace distribution (mu=0.001, scale=30.7). We corrected the xml files to specify the number of invariant sites as indicated at: https://groups.google.com/g/beast-users/c/QfBHMOqImFE. We ran two runs with shains 2×10^7^ steps long, sampling every 2000 steps and removing the initial 10 % as burn-in. We evaluated that the mixing and the estimated sample size (ESS) of the posterior and of all parameters were greater than 200, with Tracer 1.7.1 [35]. We used *logcombiner* to combine the tree files from the independent runs and the *ggtree* [36] R package to annotate and plot the trees.

Lastly, by combining results from molecular dating and the likely ancestral geographical origin from RASP, we aimed to assess for how long previously defined clades have been circulating within the country. We calculated the median and 95% HPD for MRCAs with origin in Mozambique for all clades, and we compared the average of the median years of introduction in the country among endemic and foreign clades.

### Identification of recent transmission events

Transmission was evaluated by clustering analysis from pairwise distances. We employed the strict relatedness cut-off of 5 SNPs for describing recent transmission [37], and we evaluated up to 10 thresholds for broad cluster definition. Clusters obtained from Mozambique [estimated incidence rate (IR): 551/100 000 in 2014] were compared to data from population-based studies performed in settings with different TB burden [Malawi, IR 2009 243/100 000; Valencia (Spain), IR 2015: 9.13/100 000]. From those available datasets (previously analysed under our pipeline), we chose annual data of transmission from 2009 [38] and 2015 (manuscript under revision) respectively. This was based on the largest datasets close to 2013/4 and to have comparable timespan.

### Statistical analysis

Statistical analyses were performed using the R statistical language (R version 3.5.2, The R Foundation for Statistical Computing Platform). For the phylogenetic analysis, branch lengths were extracted using the *geiger* [39] package. The *fastbaps* [40] package was applied for the BAPS algorithm. For transmission analysis, transmission events were identified by using the *ape* and *adegenet* packages. *Tidyverse, tigerstats, naniar, epiR*, *purr, broom, ggplot* and *parameters* were used to visualize, describe and analyse epidemiological data.

We aimed to explore the association of being in transmission (dependent variable) with a range of collected risk factors, such as HIV status, sex, cough or X-ray interpretation, among others (Table 1). The non-parametric Fisher’s exact test was used to identify differences in the distribution of independent variables. We hypothesized that transmission patterns would differ depending on the origin of clades (endemicity). Saturated logistic regression models were used to estimate the mutually adjusted odds ratio (OR). Covariates with *P*<0.2 were assed for adjusted analysis. Age, sex and HIV status were chosen as *a priori* related risk factors irrespective of univariate associations. A backward strategy was used to finalize the model. Afterwards, considering the large prevalence of HIV-positive patients in our dataset, and that this unbalanced distribution might confound our findings, we stratified the analysis by HIV status (Table 2).

**Table 1.** Descriptive analysis of exploratory covariates stratified by clusters of transmission

	Total (*n*=223) (%)	Unclustered (*n*=105) (%)	Clustered (*n*=118) (%)	*P*-value
**Age (years)**				0.583
<15	3 (1.3)	1 (0.9)	2 (1.7)	
15–30	71 (31.8)	34 (32.4)	37 (31.6)	
31–45	96 (43.4)	49 (46.7)	47 (40.2)	
46–60	38 (17.0)	17 (16.2)	21 (17.9)	
+60	14 (6.3)	4 (3.8)	10 (8.6)	
**Paediatric TB (<16 years)**	6 (2.7)	4 (3.7)	2 (1.8)	0.425
**Sex**				0.780
Female	79 (35.4)	36 (34.3)	43 (36.4)	
Male	144 (64.6)	69 (65.7)	75 (63.6)	
**HIV infection status**				0.642
Negative	58 (26.0)	25 (23.8)	33 (28.0)	
Positive	161 (72.2)	77 (73.3)	84 (71.1)	
Unknown	4 (1.8)	3 (2.9)	1 (0.9)	
**CD4 counts (*n*=161)^1^**				0.483
CD4 <200 cells/mm3	98 (60.9)	41 (64.1)	41 (56.9)	
CD4 >200 cells/mm3	38 (23.6)	23 (35.9)	31 (43.06)	
**Symptoms present**				
Cough	209 (93.7)	99 (94.3)	110 (93.2)	0.789
Fever	180 (80.7)	87 (82.9)	93 (78.8)	0.498
Weight loss	204 (91.8)	98 (93.3)	106 (89.8)	0.472
Night sweats	184 (82.5)	90 (85.7)	94 (79.7)	0.290
Thorax pain	73 (32.7)	37 (35.2)	36 (30.5)	0.572
**Abnormal chest X-ray^2^**	87 (52.1)	44 (55.7)	43 (49.9)	0.67
**Health Care Unit**				0.925
Calanga	5 (2.2)	2 (1.9)	3 (2.5)	
Chibucutso	4 (1.8)	1 (1.0)	3 (2.5)	
Chibututuine	12 (5.4)	6 (5.8)	6 (5.1)	
Manhiça	103 (46.4)	46 (44.2)	57 (48.3)	
Maragra	31 (14.0)	16 (15.4)	15 (12.7)	
Palmeira	28 (12.6)	14 (13.5)	14 (11.9)	
Maluana	13 (5.8)	8 (7.7)	5 (4.2)	
Malavela	10 (4.5)	4 (3.8)	6 (5.1)	
Munguine	7 (3.1)	2 (1.9)	5 (4.2)	
Taninga	9 (4.0)	5 (4.8)	4 (3.4)	
**Smoking habit (yes)**	21 (9.6)	13 (12.4)	8 (6.8)	0.172
**Alcohol use (frequent^3^)**	25 (11.4)	11 (10.7)	14 (12.1)	0.833
**History of incarceration (yes)**	22 (9.9)	15 (14.8)	7 (6.1)	0.043
TB contact (yes)^4^	46 (26.9)	26 (25.7)	20 (35.7)	0.602
Type case^5^				0.266
New	186 (89.4)	88 (88.9)	98 (89.9)	
Relapse	13 (6.1)	5 (5.5)	8 (7.3)	
Treatment after failure	1 (0.05)	0	1 (0.9)	
Treatment after LTFU	8 (3.8)	6 (6.1)	2 (1.8)	
**Pulmonary TB**	212 (95.1)	100 (95.2)	112 (94.9)	1
**Xpert MTB/RIF result**				0.336
High	73 (32.7)	33 (31.4)	40 (34.8)	
Medium	68 (30.5)	30 (28.8)	38 (33.0)	
Low	47 (21.1)	28 (26.7)	19 (16.5)	
Very low	32 (14.3)	14 (13.3)	18 (15.6)	
**Any phenotypic R^6^**	22 (9.9)	12 (11.4)	10 (8.5)	0.505
**MDR^7^**	3 (1.3)	3 (1.3)	0	0.102
**Marker of Rifampicin R**	4 (3.7)	4 (3.7)	0	0.0476
**Treatment outcomes^8^**				**0.732**
Cured	122 (68.1)	57 (69.5)	65 (67.0)	
Death	24 (13.4)	11 (13.4)	13 (13.4)	
Transferred	6 (2.7)	3 (3.7)	3 (3.1)	
Treatment completed	11 (4.9)	3 (3.6)	8 (8.2)	
LTFU^9^	6 (2.7)	2 (2.4)	4 (4.1)	
Treatment failure	10 (4.5)	6 (7.3)	4 (4.2)	
**Sublineages**				0.29
Lineage 1.1	24 (11.1)	11 (10.5)	13 (11.0)	
Lineage 1.2	57 (26.5)	21 (20.0)	36 (30.5)	
Lineage 2.2	25 (11.6)	8 (7.6)	17 (14.4)	
Lineage 3	4 (1.9)	2 (1.9)	2 (1.7)	
Lineage 4.1	26 (12.1)	13 (12.4)	13 (11.0)	
Lineage 4.3	49 (22.8)	28 (26.7)	21 (17.8)	
Lineage 4.4	9 (4.2)	6 (5.7)	3 (2.5)	
Lineage 4.10	21 (9.8)	8 (7.6)	13 (11.0)	
Endemicity^10^				0.218
Yes	54 (24.2)	21 (20.0)	33 (28.0)	
No	169 (75.8)	84 (80.0)	85 (72.0)	
				

*Fisher’s-exact test. ^1^CD4 counts not available in 25 participants; ^2^X-ray not available in 56 participants; ^3^more than 2/3 times per week; ^4^six cases did not provide information; ^5^15 responses were not available; ^6^R: resistant; ^7^MDR: multidrug resistant TB; ^8^information on TB outcomes not available in 44 cases; ^9^LTFU: lost-to-follow-up; ^10^endemicity defined as strains belonging to BASP clades that were estimated to have an MRCA from Mozambique (see Methods).

**Table 2.** Multivariable logistic regression results on the association of endemicity with clustering as the outcome variable, for the entire cohort and after stratification by HIV status

	aOR^1^ transmission cluster	aOR^3^ transmission cluster (PLHIV^4^) (95 % CI)	*P*-value	aOR transmission cluster (HIV-) (95 % CI)	*P*-value
	(*n*=213)^2^	*n*=157		*n*=55	
Endemic	1.42 (0.75;2.75)	2.19 (1.08; 4.59)	0.033	0.28 (0.05;1.23)	0.102
Non-endemic	1	1		1	

^1^aOR: best fitted multivariable regression model adjusted by sex, age group and HIV status; ^2^total of cases excluding mixed infections (*n*=8) and missing values (two HIV status unknown), ^3^aOR: best fitted multivariable regression model adjusted by sex and age, calculated after stratifying by HIV status; ^4^PLHIV: people living with HIV.

## Results

### Overall population structure of MTBC strains in Manhiça district

**Fig. 1.**
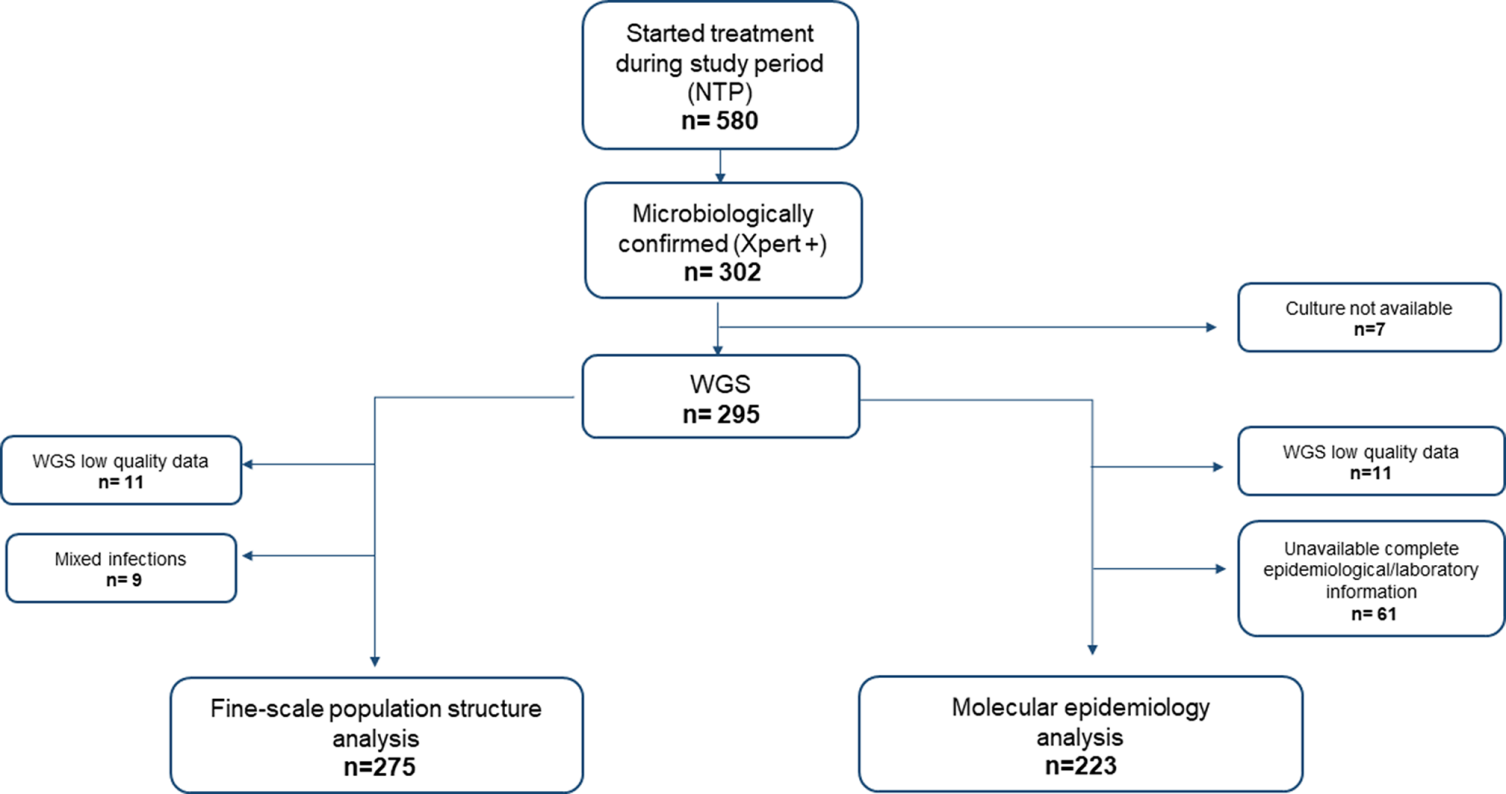
Study flowchart. NTP: National Tuberculosis Programme; Xpert+: samples that tested positive by Xpert MTB/RIF; WGS: whole genome sequencing.

During the study period, 580 patients started treatment through the NTP in the district; 74.3 % (431/580) of them were PLHIV (close to our 72.2 %. Table 1), and 164 out of 431 were on antiretroviral therapy (ART) (38.1 % of PLHIV). From those 580 patients notified, 302 were microbiologically confirmed by Xpert, although seven of those strains were not available for WGS due to poor quality of cultures. After exclusions (Fig. 1), 275 strains were included in the analysis on population structure.

**Fig. 2.**
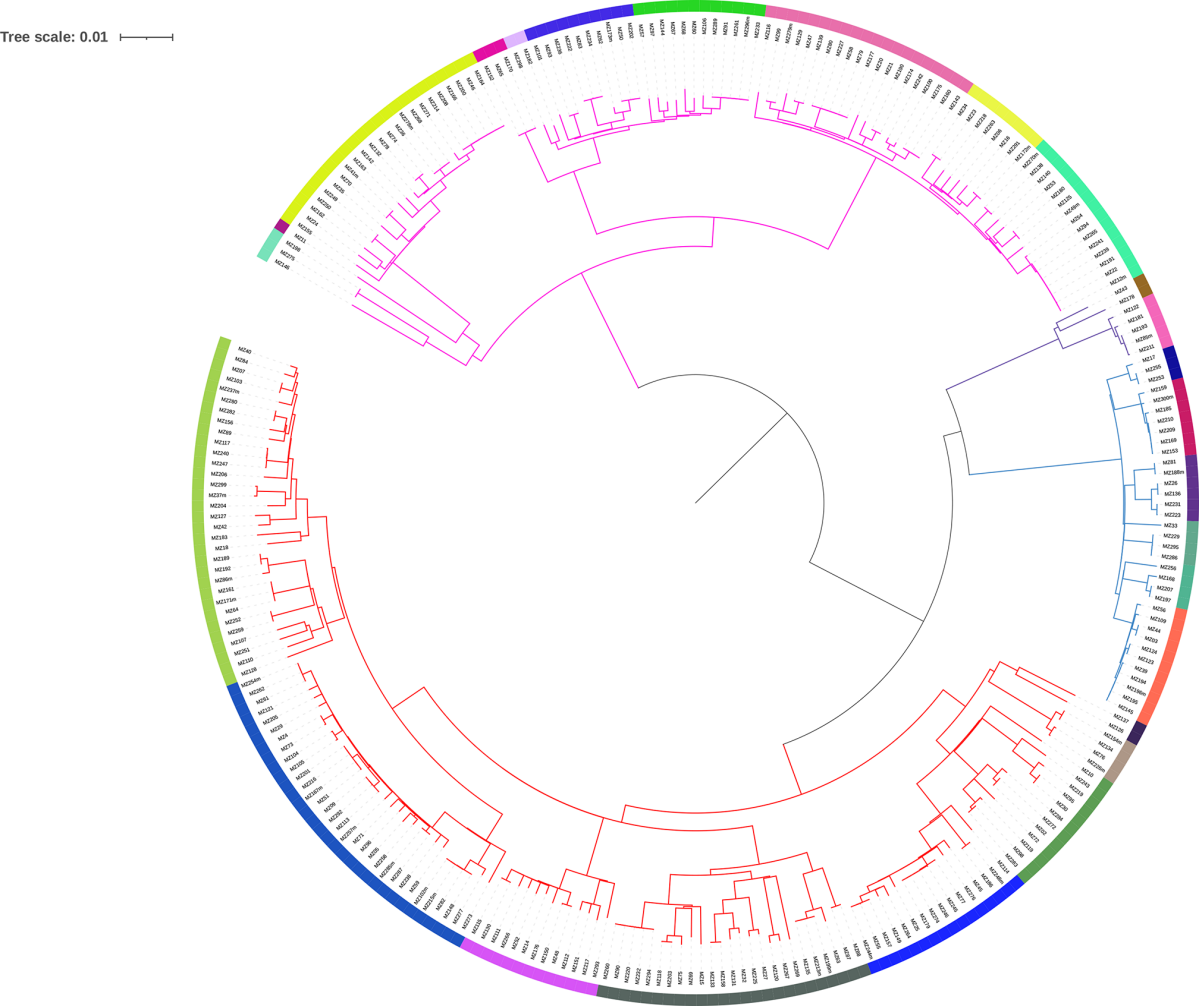
Phylogenetic tree, 275 strains. Lineages are represented in branch colours (pink for L1, blue for L2, purple for L3 and red for L4). Twenty-five BAPS groups are denoted by different colours in the outer ring of the phylogeny.

Overall, when reconstructing the phylogenetic tree (Fig. 2), most isolates were assigned to L4 (49.5%, 136/275). The L4.3.4 (LAM) sub-lineage was the most common within L4 (23.3%, 64/275), although at this level the most prevalent sublineage was L1.2 (25.4 %, 70/275). Beijing L2 were classified as L2.2.1, representing 12.7 % (35/275) of the total population (Table S1, available with the online version of this article). Additionally, nine samples were determined as mixed infections.

### SNP-threshold approach to evaluate transmission events Fig. 3

**Fig. 3.**
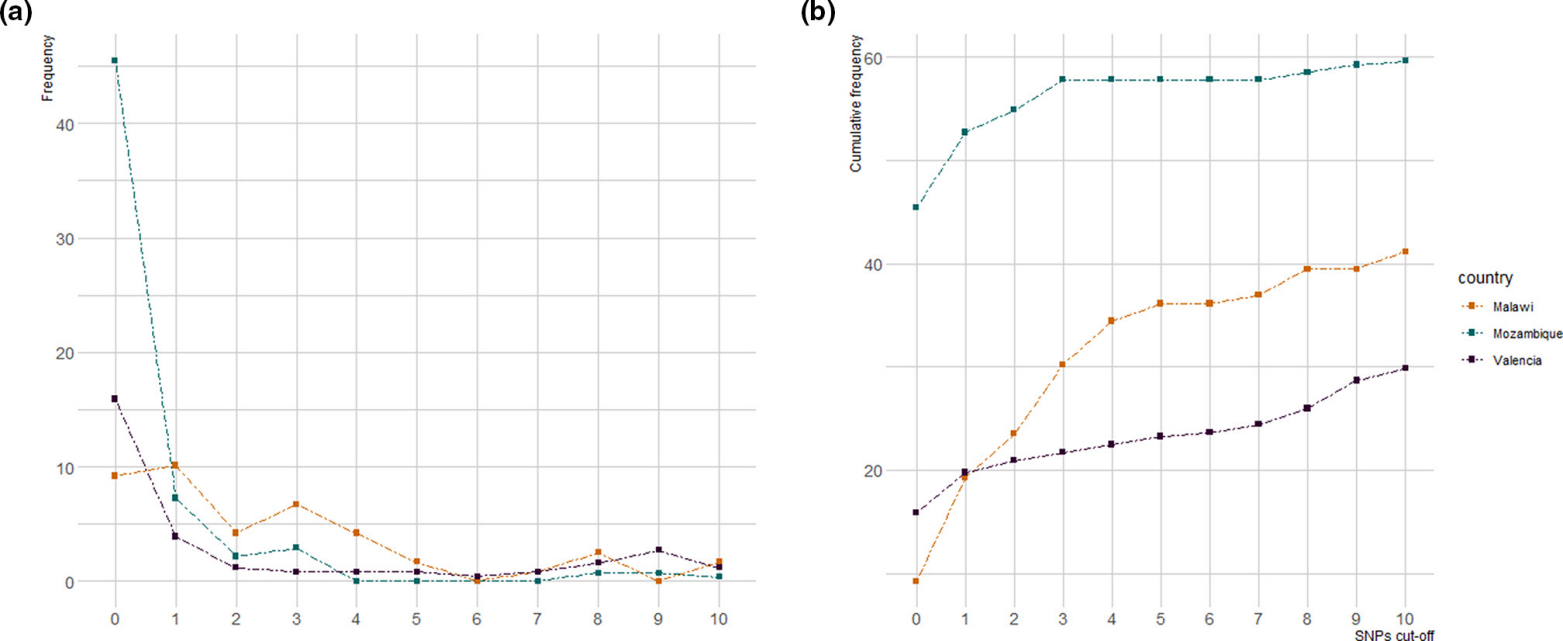
Proportion of strains linked by pairwise distance from 0 to 10 SNPs, using data from three different datasets. (**a**) Frequency of samples found at each pairwise distance. (**b**) Cumulative frequency (%) of isolates in transmission at pairwise distance from 0 to 10 SNPs. Colours: the blue line represents data from Mozambique (2013–2014), orange from Malawi (2009) and purple from Valencia (2015).

Clustering analysis revealed that 57.8 % of strains (159/275) occurred in a potential transmission cluster, applying a cut-off pairwise distance of 5 SNPs. To evaluate the ‘transmission profile’ we compared genetic distances up to 10 SNPs with previously published data from Malawi (2009) and Valencia [19] (see Methods). The distribution of pairwise distances revealed that, in all three regions, a large proportion of cases were in transmission, although patterns differed from one population to another, being greater in Mozambique (64.7 %) compared to Malawi (41.2 %) and Valencia (29.8 %) (Fig. 3).

While TB transmission seemed continuous for all three sites, the burden of very recent spread was significantly higher in Mozambique: 45.5 % (125/275) of strains shared an identical genotype (0 SNPs genetic distance), whereas only 9.2 % (11/119) and 15.9 %(41/246) were identified for Malawi and Valencia, respectively.

### Fine-scale population structure of MTBC

**Fig. 4.**
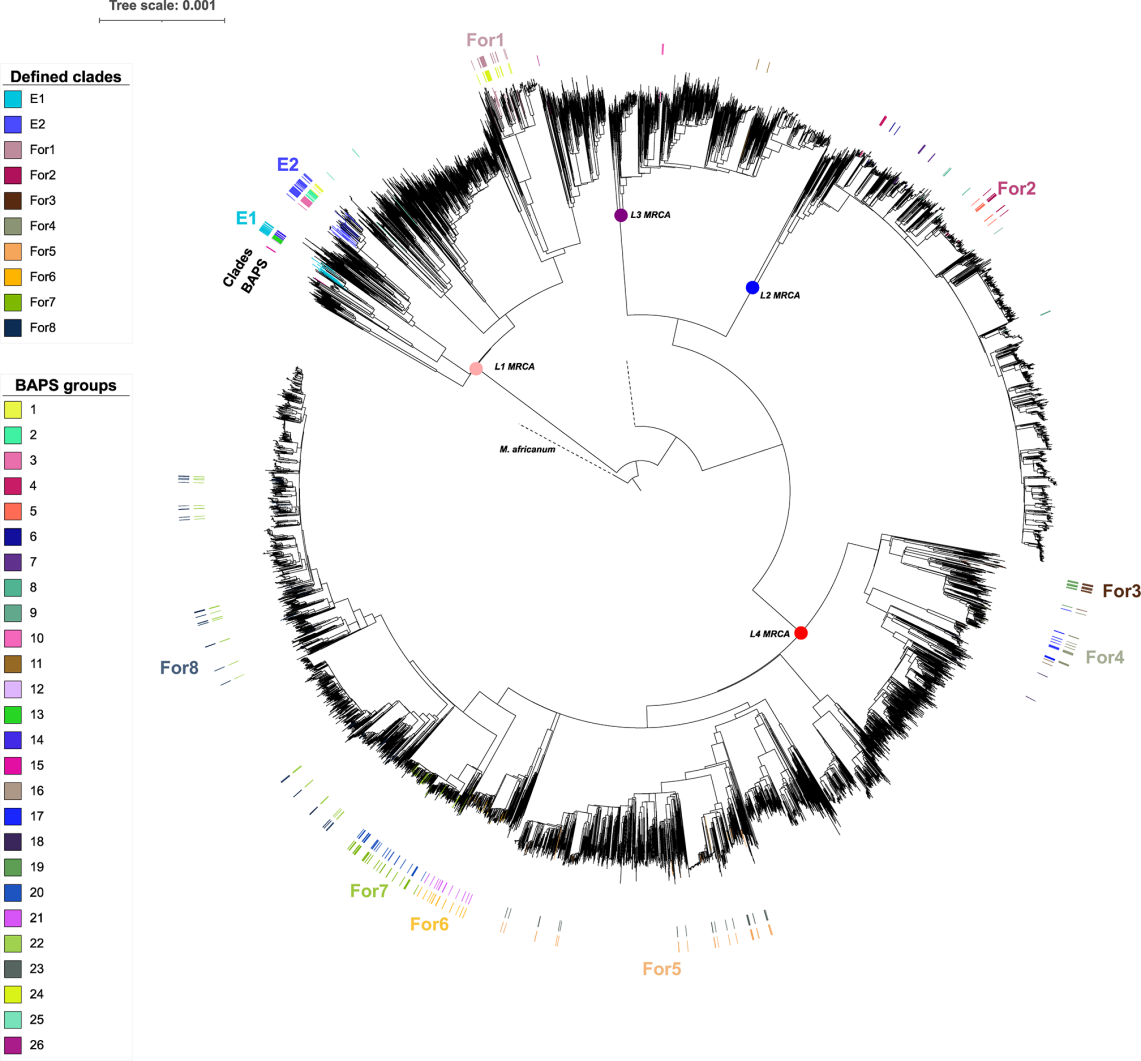
Phylogeographic approach. We reconstructed a phylogeny by combining isolates from Mozambique (MZ) and 8263 genomes representative of the MTBC global genetic diversity. This was used as input to infer the origin of BAPS ancestors by RASP (R package). Following defined criteria (see Methods), we classified two large endemic clades (E) and eight foreign clades (For: non-endemic). The rest of the BAPS groups could not be classified as endemic or not. MRCA: most recent common ancestor.

By applying the BAPS algorithm, we recognized 25 groups of strains that shared high genomic similarity at level 2 of the clustering hierarchy: nine groups from L1, six L2, two L3 and eight L4 (one strain did not assemble) (Fig. 2). These groups were mapped in a global phylogeny (Fig. 4) and, through the use of RASP, we inferred the probable geographical origin of the MRCA for each group (see Methods).

BAPS groups 1, 2, 3, 12, 13 and 14 were enriched in Mozambique isolates. BAPS groups 1, 2 and 3, and 12, 13 and 14 formed two larger groups which shared an MRCA with origin in the country (probability of 80%). Therefore, we merged those BAPS into two clades, and considered them ‘likely endemic’ or ‘local’ in our study setting (E1 and E2, 67/275, 24.4%) (Table S2). Eight clades were defined as probably ‘non-endemic’ candidates (164/275, 59.6%), and 11 did not reach the defined criteria to be classified (see Methods). All strains categorized as endemic belonged to L1.2.

**Fig. 5.**
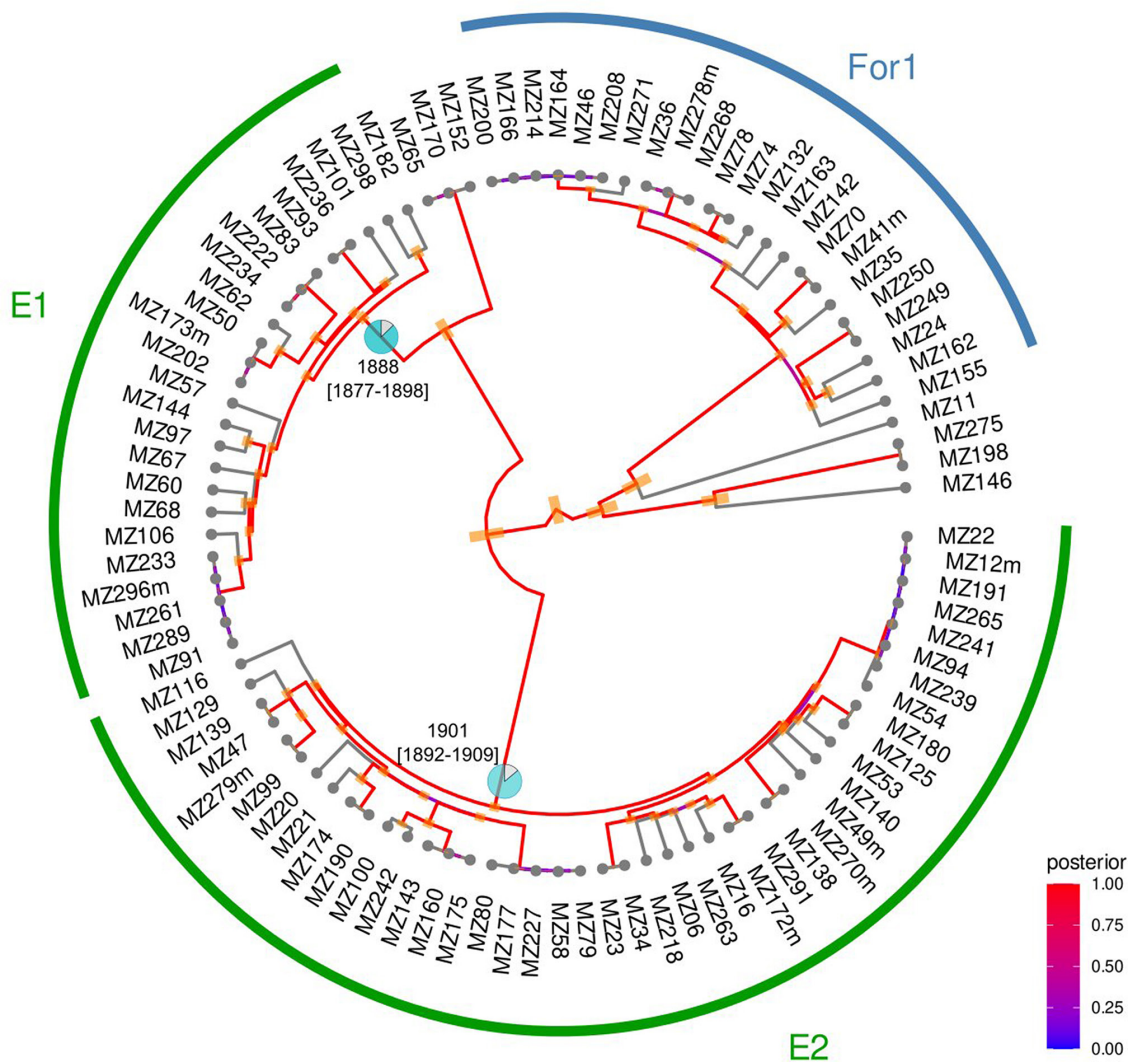
Endemic and non-endemic clades from L1 with BEAST dating results for the MRCA with origin in Mozambique. Pie charts represent the probability that the geographical origin of the ancestor was Mozambique (MZ). The 95 % HPD is drawn as orange intervals and refers to the ‘confidence interval’ for each ancestor estimated date. The posterior indicates the probability distribution over the parameter state space. This refers to the strength of the calculated temporal parameter. The red colour (posterior close to 1) reveals strong confidence on estimations performed for the date under the model of evolution applied. E: endemic; For: foreign (non-endemic); 95 % highest posterior density.

We then inferred the age of probable endemic and non-endemic clades (Table S3). We found that, for those considered as ‘likely endemic’, MRCAs located in Mozambique dated further back in time than in non-endemic ones. The mean of the median time when those local clades began to circulate in the country was 1894 [IQR 1887.7; 1900.6], whereas for non-endemic clades, it was estimated to be 2009 [IQR 1876.9; 2014.0] (Fig. 5, Figs S4.1 and 4.2).

### Impact of collected risk factor and endemicity on transmission

Of those patients with WGS data, 223 isolates had laboratory and epidemiological data available (Fig. 1). They were then considered to investigate potential covariates associated with clustering, by using a restrictive cut-off of pairwise distance at 5 SNPs, in order to evaluate very recent transmission (see Methods).

The median age of patients was 35 years [IQR 28.2; 45.0], 35.4 % (79/223) were female and the majority (72.2 %, 161/223) were PLHIV. The median CD4+ count was 145.5 [49.9; 321.8] cells mm^–3^. The most common symptom was coughing, which was present in 93.7 % of cases (209/223). Sputum smear for acid-fast bacilli was positive in 65.0 % (145/223) and 52.1 % of participants had a pathological chest X-ray (CXR) (87/167 available). In this subset of data, 52.9 % of isolates (118/223) were included in a cluster. The distribution of strains in recent transmission did not vary by sex, age, HIV status, symptoms, type of TB, different areas of the district or sublineage (*P*>0.05). Although none of the resistant strains were clustered, we were unable to explore this association further due to the limited number of isolates. Univariate exploration of other sociodemographic and microbiological variables is displayed in Table 1.

Based on the hypothesis that local genotypes might be linked to transmission, we explored the association between ‘endemicity’ and clustering. Overall, 24.2 % (54/223) of strains were classified as ‘likely endemic’ (hereinafter endemic). Of these, 83.3 % (45/54) were isolated in PLHIV, whereas just 16.7 % (9/54) were found in HIV-negative individuals. Of the total number of isolates that occurred in a cluster, 28 % were classified as endemic (33/118) and 20 % as non-endemic (21/105). The results of the best fitted logistic regression model are shown in Table 2. To eliminate any potential confounding effect caused by the different distributions of HIV/TB coinfection in our cohort, subsequent stratification revealed that PLHIV who were infected with endemic strains had more than a two times higher odds of being in recent transmission, regardless sex and age (2.11, 95 % CI [1.04;4.45], *P*=0.04), and that this association disappeared among HIV-negative individuals.

Lastly, in light of those results, we investigated whether the degree of immunosuppression, measured through CD4 levels, might shape the distribution of endemic clades. We found that although the proportion of endemic strains increased with a decrease in CD4 counts, this was not significant (*P*=0.134) (Fig. S5).

## Discussion

High-resolution WGS data on MTBC population structure and transmission patterns are scarce in Mozambique [13, 41], and similar to the situation for most high-burden countries. To provide comprehensive insight into the molecular epidemiology of MTBC in our area, we have conducted the first population-based study using WGS data. Cluster analysis revealed a high profile of recent transmission. We also found two major ‘likely endemic’ clades that were associated with recent transmission among PLHIV. These clades were dated back to the end of the 19th century.

When pairwise distances were compared to 1 year of data from Malawi and Spain, the profiles revealed that the highest proportion of strains involved in transmission was in Mozambique (64.7%), and was also higher than that found in other high-burden settings (e.g. Liberia 39%, using a cut-off of 12 SNPs) [42], and reported in Malawi for 15 years (31%) [38]. Although high rates of ongoing transmission were expected [3, 43 ], almost half of the strains were connected by 0 SNPs. Assuming a molecular clock of 0.3–0.5 SNPs per year, this would translate to very recent transmission events [44] and fast rates of progression to active TB. Importantly, this proportion might be even underestimated as 1 year population analyses are not sufficient to reveal the extent of recent transmission.

Interrupting ongoing spread is a critical public health intervention to bring down the current TB burden. The present data reveal the suboptimal control of the epidemic in our setting and reinforce the need to explore the local scenario. Most of the participants were severely ill at the time of diagnosis, which might support previous studies describing low awareness of disease [45, 46] and/or the existence of barriers in accessing medical care in our setting [47]. Besides, programmatic active-case finding activities have been described as inconsistent in Mozambique, failing to reveal hidden cases and breaking the transmission net [48]. As recently stated in the Global Fund report [49], there is a need to strengthen the cascade of care by the identification of patients and adherence to treatment. In our cohort of microbiologically confirmed cases (highly transmitters), 20 % of participants had contact with a TB case, although they sought health care because they were symptomatic (presumptive), revealing a lack of active screening activities to identify patients at early stages.

The globally spread L4 was the most prevalent in our region, in agreement with previous studies [10], although at the sublineage level, L1.2 was the most frequent. This finding aligns with the global distribution of L1, concentrated around the Indian Ocean and that has been considered to spread from South Asia as a result of old migration pathways [50]. To define clades specific to Mozambique, i.e. ‘probably endemic’, we applied a detailed, fine-scale population structure approach that allowed us to define two major endemic clades, both belonging to the L1.2 sub-lineage. Consistent with the finding that they are likely to be endemic in our study setting, dating estimates confirmed that they would have been circulating in Mozambique for longer than non-endemic clades.

The long-term presence of these clades in Mozambique made us question whether local strains might have coevolved within the sympatric host population, as seen elsewhere [51]. Thus, we tested the likelihood that endemic clades, as well as other collected covariates, could be related to a higher probability of recent spread using a pairwise distance of 5 SNPs as a threshold [37]. Stratification by HIV status revealed that those classified as probably endemic presented higher odds of being responsible for recent transmission, but just among HIV-positive patients. This can be understood because 83 % of the endemic strains were found in this population, and two-thirds of them were found in transmission clusters.

The extent to which TB/HIV coinfection influences the structure of the MTBC population and the efficacy of transmission is currently under debate [52, 53]. On the one hand, studies evaluating MTBC infectiousness among PLHIV are widely heterogeneous [53, 54]. Historically it has been argued that HIV decreases transmission, due to extrapulmonary TB (EPTB), lower cavitary lesions or lower sputum bacillary load, among others [52, 55]. Nevertheless, recent studies state that HIV-seropositive patients are as infectious as seronegative patients when they present cavitary disease or have a positive bacilloscopy [53, 56]. Furthermore, among PLHIV, most cases of active TB are the result of new infections [57]. Therefore, for our setting, the large proportion of strains in recent spread may be due to the fact that half of PLHIV participants were severely immunosuppressed (highly susceptible to developing disease), only 38 % of those registered as HIV/TB coinfected were on ART, most of them were microbiologically confirmed and just 5 % of them had extrapulmonary disease.

A previous study on coevolution demonstrated increased transmission between sympatric linage–host associations [11], although in that case, transmission was disrupted by HIV coinfections. This finding contrasts with our results: increased transmission between sympatric strain–host associations, but just among PLHIV. Yet, the interdependence of both epidemics in high-burden settings needs to embrace socioeconomical determinants that may shape the circulation and spread of MTBC and confound results [55]. Further studies on how HIV may influence sympatric associations in high-burden areas are needed. Alternatively, regardless of HIV status, and according to a recent phylogeographic study [58], L1 would act as a specialist genotype, geographically restricted and adapted to the local population, which would constitute a reservoir and lead to these strains being endemic. Limitations for understanding this interplay include the fact that the ancestry of individuals involved in this study is not known and that transmission estimates are based on a 1 year sampling window. Although this may have limited our results, given the fast progression of the disease in PLHIV and the particularity of our dataset (45.5 % of strains has pairwise distance of 0 SNPs), 1 year's data may indeed provide a hint of what is happening in our region.

Lastly, we cannot ignore that host-related factors must be considered when interpreting genomic data. Information on the interaction between host and pathogen genotypes is still scarce and few genotype–genotype association studies are available [7]. Since the adaptive immune response is an essential mechanism for host recognition and control of MTBC [59], we hypothesize that the interaction of HIV, MTBC and human populations may be more complex in countries where the two epidemics collide with an uncertain impact on TB transmission. We highlight the need for population-based genome-to-genome association studies, including MTBC, human and HIV genotype combined with sociodemographic data that could potentially confound results.

## Conclusion

Overall, our results reveal the population structure of MTBC in a high TB and HIV burden setting. We found an unexpected pattern of transmission, with the majority of isolates being in genomic cluster. This suggests the uncontrolled TB spread and high rates of progression to active disease. We also identified endemic clades that were estimated to be circulating in the country for more than a century and that were responsible for recent transmission among PLHIV.

## Supplementary Data

Supplementary material 1
