# Supplementary material for: Fine-grain population structure and transmission patterns of Mycobacterium tuberculosis in southern Mozambique, a high TB/HIV burden area
Source: Microb Genom. 2022 Jul 5;8(7):mgen000844. doi: 10.1099/mgen.0.000844 (PMC9455694; doi:10.1099/mgen.0.000844)

Supplementary S1. Table 1. Distribution of lineages and sublineages overall and stratify by clustering and likely endemic clades

Lineage	n= 275	(%)		
1	97	35.3		
2	35	12.7		
3	7	2.5		
4	136	49.5		
**Sublineages**				
L1.1	27	9.8		
L1.2	70	25.4		
L2.2.1	35	12.7		
L3	7	2.6		
L4.1	34	12.4		
L4.3.4	64	23.3		
L4.4	13	4.7		
L4.10	25	9.1		
**Sublineages[1]**	**Unclustered (116)**	**(%)**	**Clustered (159)**	**(%)**
L1.1	10	8.6	17	10.7
L1.2	25	21.5	45	28.3
L2.2	9	7.8	26	16.3
L3	4	3.4	3	1.9
L4.1	16	13.8	18	11.3
L4.3	32	27.6	32	20.1
L4.4	9	7.8	4	2.5
L4.10	11	9.5	14	8.8
**Sublineages[2]**	**Non-endemic (208)**	**(%)**	**Endemic (67)**	**(%)**
L1.1	27	13.0	0	0
L1.2	3	1.4	67	100
L2.2	35	16.8	0	0
L3	7	3.4	0	0
L4.1	34	16.3	0	0
L4.3	64	30.8	0	0
L4.4	13	6.2	0	0.0
L4.10	25	12.0	0	0.0

[1] p-value=0.09 ; [2] p-value<0.001 Fischer-exact test

Supplementary S2. Table 2. Summary of BAPs groups

BAPS group	Type of clade	lineage	clade name	Number of strains
1	endemic	L1	E2	8
2	endemic	L1	E2	15
3	endemic	L1	E2	20
4	unknown	L2	Unk4	7
5	non-endemic	L2	For2	11
6	unknown	L2	Unk5	3
7	unknown	L2	Unk6	6
8	unknown	L2	Unk7	4
9	unknown	L2	Unk8	4
10	unknown	L3	Unk10	5
11	unknown	L3	Unk11	2
12	endemic	L1	E1	2
13	endemic	L1	E1	12
14	endemic	L1	E1	10
15	unknown	L1	Unk3	3
16	unknown	L4	Unk10	4
17	foreign	L4	For4	16
18	unknown	L4	Unk9	2
19	non-endemic	L4	For3	12
20	non-endemic	L4	For7	32
21	non-endemic	L4	For6	13
22	non-endemic	L4	For8	33
23	non-endemic	L4	For5	25
24	non-endemic	L1	For1	23
25	unknown	L1	Unk1	3
26	unknown	L1	Not grouped	1

Footnotes: E: Endemic; For: Foreign (non-endemic); Unk: Unknown, not possible to be classified

Supplementary S3. Table 3. Median time to the Mozambican Most Recent Common C (MRCA) for each clade and calculation of mean´s median times, by BAPS type (endemic(E) or foreign (For))

			Estimated time of the Mozambican MRCA	
**BAPS type**	**Lineage**	**Clade**	**Median time (years)**	**95% HPD[1] (years)**
endemic	L1	E1	1887.73	[1877.4 – 1898]
endemic	L1	E2	1900.61	[1891.9 – 1909.1]
**Mean Endemic**			**1894.17**	**Range [1887.7 – 1900.6]**
foreign	L1	For1-1	1932.45	[1923.6 – 1941.1]
foreign	L1	For1-2	2014	
foreign	L1	For1-3	2014	
foreign	L1	For1-4	2014	
foreign	L1	For1-5	2013.57	[2012.2 – 2014]
foreign	L1	For1-6	2014	
foreign	L1	For1-7	2013.62	[2012.5 – 2014]
foreign	L2	For2-1	2013.22	[2010.5 – 2014]
foreign	L2	For2-2	1982.07	[1966.5 – 1995.4]
foreign	L2	For2-3	2014	
foreign	L4	For3-1	2012.76	[2008 – 2014]
foreign	L4	For3-2	2014.00	
foreign	L4	For3-3	2014.00	
foreign	L4	For3-4	2014.00	
foreign	L4	For3-5	2014.00	
foreign	L4	For3-6	2014.00	
foreign	L4	For3-7	2012.89	[2009.2 – 2014]
foreign	L4	For3-8	2012.82	[2009.2 – 2014]
foreign	L4	For4-1	2008.84	[2002.7 – 2013.3]
foreign	L4	For4-2	2005.50	[1998.2 – 2011.3]
foreign	L4	For4-3	2012.79	[2008.7 – 2014]
foreign	L4	For4-4	2014	
foreign	L4	For4-5	2014	
foreign	L4	For4-6	2014	
foreign	L4	For4-7	2014	
foreign	L4	For4-8	2014	
foreign	L4	For5-1	1917.98	[1885.4 – 1947.3]
foreign	L4	For5-2	2011.43	[2006.2 – 2014]
foreign	L4	For5-3	2008.65	[2002.5 – 2013.1]
foreign	L4	For5-4	2012.57	[2007.8 – 2014]
foreign	L4	For5-5	2010.99	[2005.4 – 2014]
foreign	L4	For5-6	2011.14	[2005.4 – 2014]
foreign	L4	For5-7	2014	
foreign	L4	For5-8	2014	
foreign	L4	For5-9	2014	
foreign	L4	For5-10	2014	

foreign	L4	For5-11	2014	
foreign	L4	For5-12	2014	
foreign	L4	For5-13	2014	
foreign	L4	For5-14	2014	
foreign	L4	For5-15	2014	
foreign	L4	For6-1	2013.07	[2009.1 – 2014]
foreign	L4	For6-3	2014	
foreign	L4	For6-4	2014	
foreign	L4	For6-5	2014	
foreign	L4	For6-6	2014	
foreign	L4	For6-7	2014	
foreign	L4	For6-8	2014	
foreign	L4	For6-9	2014	
foreign	L4	For6-10	2014	
foreign	L4	For6-11	2014	
foreign	L4	For7-1	1998.36	[1987.1 – 2007.2]
foreign	L4	For7-2	2008.97	[2002.8 – 2012.8]
foreign	L4	For7-3	2008.33	[2001.7 – 2012.7]
foreign	L4	For7-4	2007.93	[2000.7 – 2012.6]
foreign	L4	For7-5	1876.90	[1852.9 – 1899.6]
foreign	L4	For7-6	2011.74	[2007 – 2013.8]
foreign	L4	For7-7	2008.95	[2002.6 – 2013.1]
foreign	L4	For7-8	2012.72	[2007.9 – 2014]
foreign	L4	For7-9	2014	
foreign	L4	For7-10	2014	
foreign	L4	For7-11	2014	
foreign	L4	For7-12	2014	
foreign	L4	For7-13	2014	
foreign	L4	For7-14	2014	
foreign	L4	For7-15	2014	
foreign	L4	For7-16	2014	
foreign	L4	For7-17	2014	
foreign	L4	For7-18	2014	
foreign	L4	For8-1	1988.60	[1973.5 – 2000.9]
foreign	L4	For8-2	2004.50	[1995.7 – 2010.5]
foreign	L4	For8-3	2011.88	[2007.2 – 2014]
foreign	L4	For8-4	2012.88	[2008.8 – 2014]
foreign	L4	For8-5	2013	[2009.4 – 2014]
foreign	L4	For8-6	2011.33	[2005.9 – 2014]
foreign	L4	For8-7	2012.59	[2014 – 2012.6]
foreign	L4	For8-8	2014	
foreign	L4	For8-9	2014	
foreign	L4	For8-10	2014	
foreign	L4	For8-11	2014	
foreign	L4	For8-12	2014	
foreign	L4	For8-13	2014	

foreign	L4	For8-14	2014	
foreign	L4	For8-15	2014	
foreign	L4	For8-16	2014	
foreign	L4	For8-17	2014	
foreign	L4	For8-18	2014	
foreign	L4	For8-19	2014	
foreign	L4	For8-20	2014	
foreign	L4	For8-21	2014	
foreign	L4	For8-22	2014	
foreign	L4	For8-23	2014	
**mean Foreign**			**2008.92**	**Range [1876.9 – 2014]**

Footnote: [1] **95% HPD: Highest Posterior Density :** the smallest interval that includes 95% of the posterior probability and refers to the 'confidence interval' for each ancestor estimated dates.

Supplementary S4.

Figure 4.1 BEAST tree for strains belonging to lineage 3

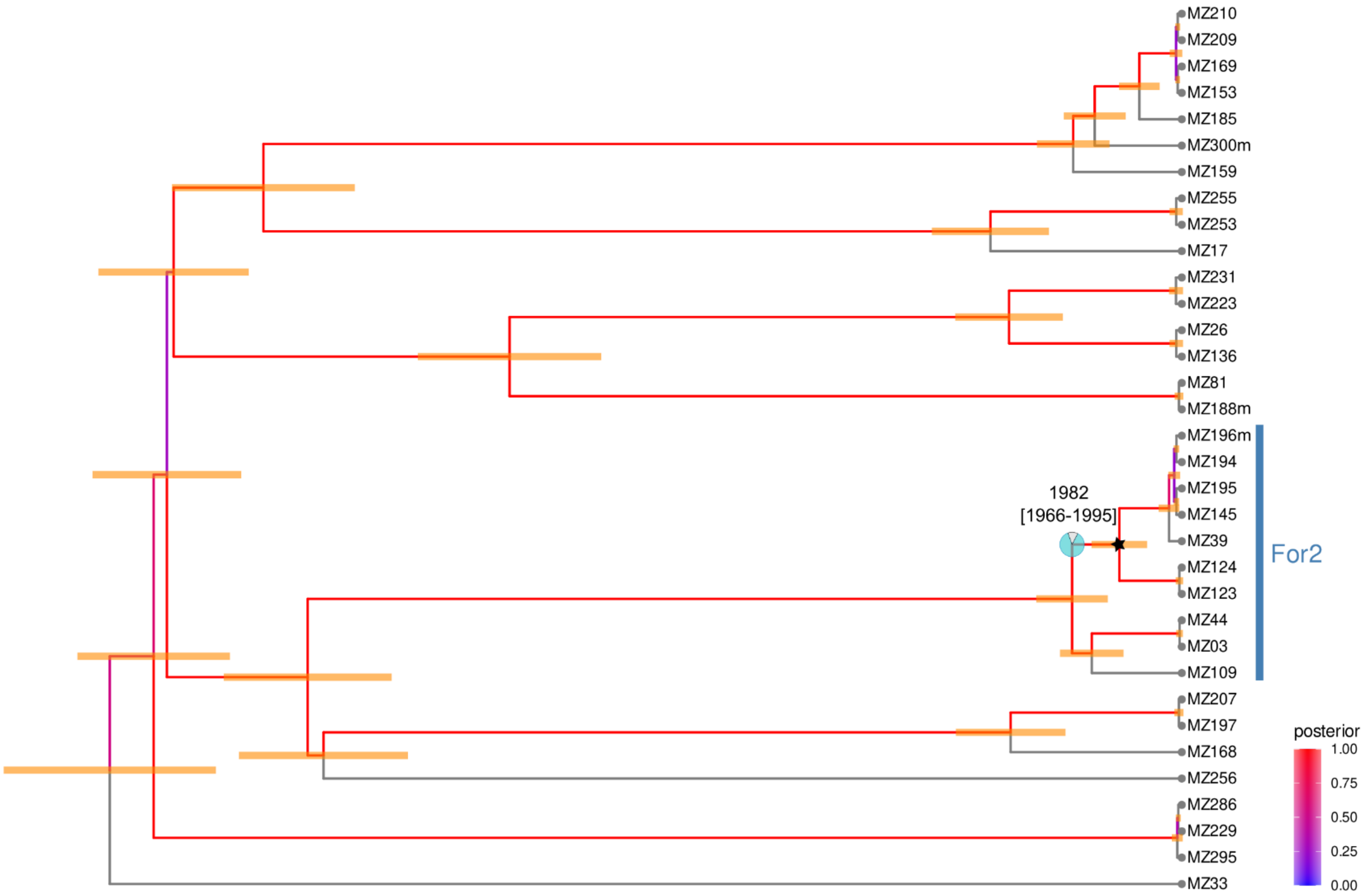

L3 BEAST tree. Dating results for the MRCA with origin in Mozambique are displayed. Pie charts represents the probability that the geographical origin of the ancestor was Mozambique (MZ). The 95% Highest Posterior Density (HPD) is drawn as orange intervals and refers to the 'confidence interval' for each ancestor estimated dates. The posterior indicates the probability distribution over the parameter state space. This refers to the strength of the calculated temporal parameter. The red colour (posterior close to one) reveals high strong confidence on estimations performed for the date under the model of evolution applied.

Footnotes: E: Endemic; For: Foreign (non-endemic); 95% Highest Posterior Density

Figure 4.2. BEAST tree lineage 4

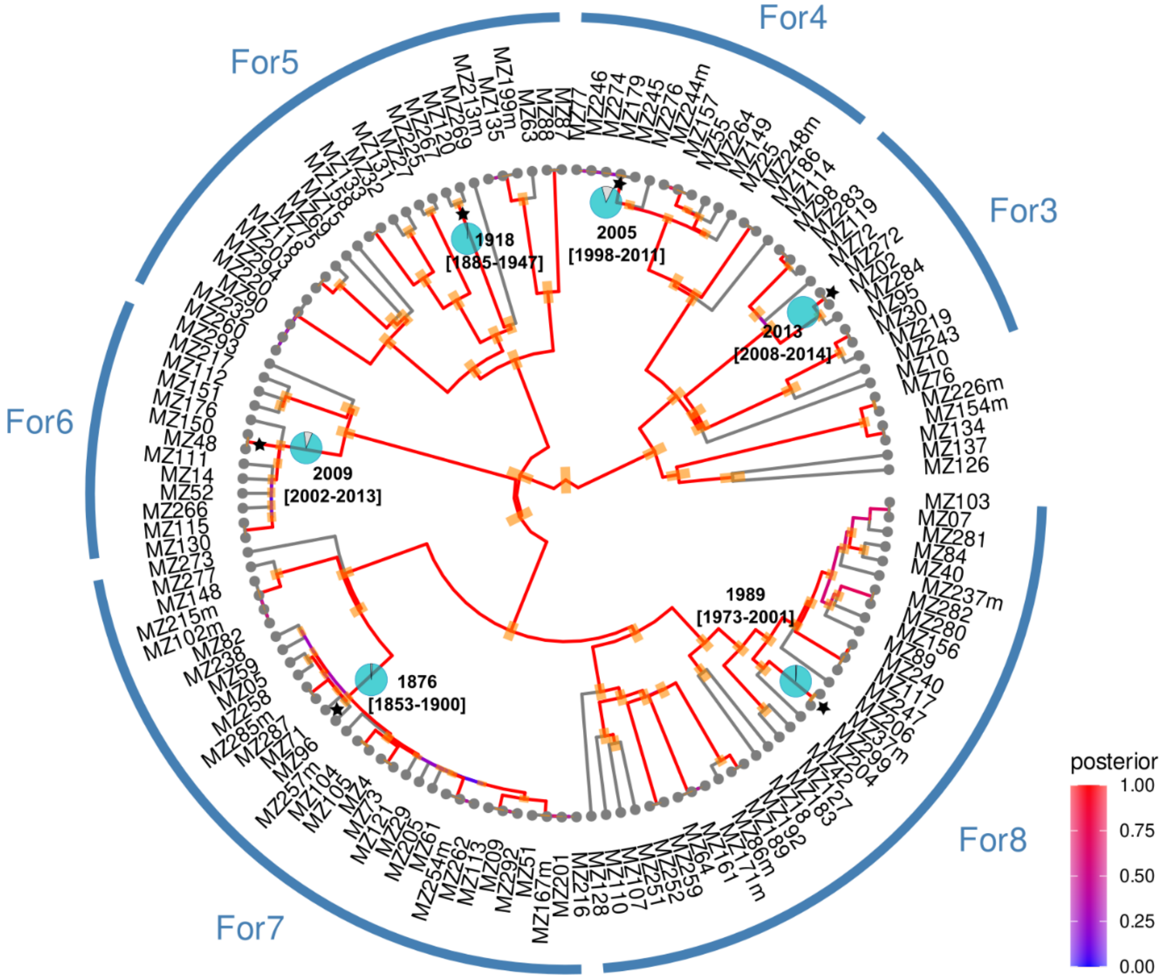

L4 BEAST tree. Dating results for the MRCA with origin in Mozambique are displayed. Pie charts represents the probability that the geographical origin of the ancestor was Mozambique (MZ). The 95% Highest Posterior Density (HPD) is drawn as orange intervals and refers to the 'confidence interval' for each ancestor estimated dates. The posterior indicates the probability distribution over the parameter state space. This refers to the strength of the calculated temporal parameter. The red colour (posterior close to one) reveals high strong confidence on estimations performed for the date under the model of evolution applied.

Footnotes: E: Endemic; For: Foreign

Supplementary S5. Graphical representation of relation between immunity status of participants and endemicity and data on the proportion of endemic and non-endemic clades stratified by CD4 counts PLHIV: People living with HIV

	**Endemic (%)** [2]	**Non-endemic (%)**	**p-value**[3]
**(n=188)** [1]			0·130
**>200 cc/mm3**	14 (25·9)	40 (74·1)	
**<200 cc/mm3**	25 (31·6)	54 (68·4)	
**Negative**	9 (16·4)	46 (83·6)	

Footnote: [1]excluding coinfections (8), HIV-status missing (2) and CD4 missing (25); [2]row percentages; [3] Fisher´s exact tes

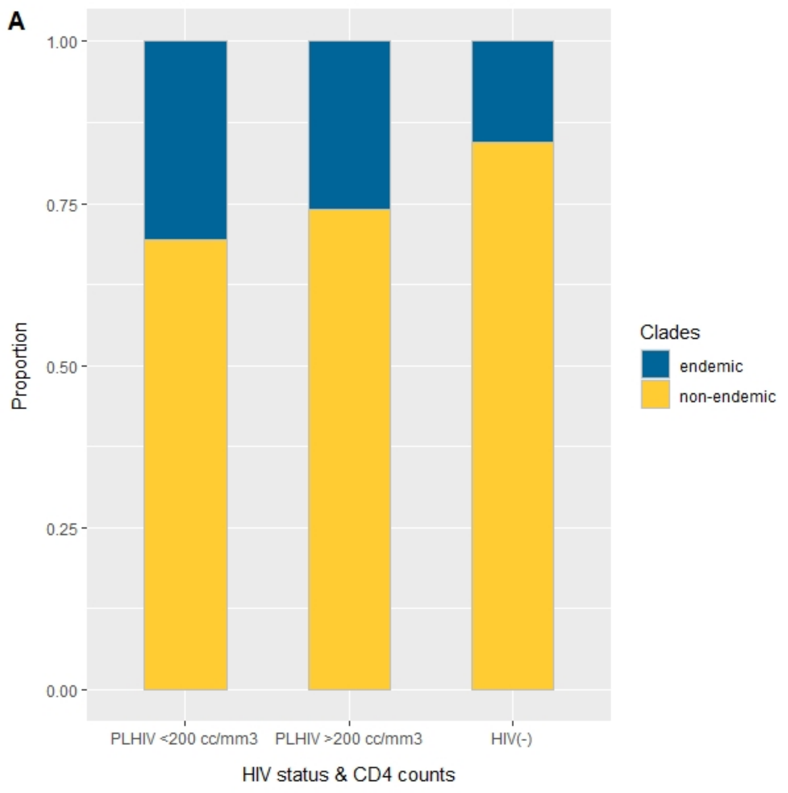

Supplement: Supplementary material 1 [file mgen-8-844-s001.pdf]
